# Unraveling Comparative Anti-Amyloidogenic Behavior of Pyrazinamide and D-Cycloserine: A Mechanistic Biophysical Insight

**DOI:** 10.1371/journal.pone.0136528

**Published:** 2015-08-27

**Authors:** Sumit Kumar Chaturvedi, Nida Zaidi, Parvez Alam, Javed Masood Khan, Atiyatul Qadeer, Ibrar Ahmad Siddique, Shamoon Asmat, Yusra Zaidi, Rizwan Hasan Khan

**Affiliations:** 1 Interdisciplinary Biotechnology Unit, Aligarh Muslim University, Aligarh, 202002, India; 2 Department of Zoology, Aligarh Muslim University, Aligarh, 202002, India; University of Akron, UNITED STATES

## Abstract

Amyloid fibril formation by proteins leads to variety of degenerative disorders called amyloidosis. While these disorders are topic of extensive research, effective treatments are still unavailable. Thus in present study, two anti-tuberculosis drugs, i.e., pyrazinamide (PYZ) and D-cycloserine (DCS), also known for treatment for Alzheimer’s dementia, were checked for the anti-aggregation and anti-amyloidogenic ability on Aβ-42 peptide and hen egg white lysozyme. Results demonstrated that both drugs inhibit the heat induced aggregation; however, PYZ was more potent and decelerated the nucleation phase as observed from various spectroscopic and microscopic techniques. Furthermore, pre-formed amyloid fibrils incubated with these drugs also increased the PC12/SH-SY5Y cell viability as compare to the amyloid fibrils alone; however, the increase was more pronounced for PYZ as confirmed by MTT assay. Additionally, molecular docking study suggested that the greater inhibitory potential of PYZ as compare to DCS may be due to strong binding affinity and more occupancy of hydrophobic patches of HEWL, which is known to form the core of the protein fibrils.

## Introduction

Protein misfolding leading to formation of amyloid aggregates has been implicated in a variety of debilitating neurological and non-neurological disorders such as Alzheimer's, Parkinson's, Huntington's, Prion and Systemic amyloidosis diseases [1,2,3]. Despite the dissimilarity in the amino acid sequences and biochemical properties, almost all the proteins have shown tendency to undergo amyloid fibrillation on its own or under external altered conditions like temperature, pH, ionic strength and denaturants etc. [4,5,6,7]. However, the mechanism that leads to protein misfolding and aggregation is not common for all proteins [8]. In recent years, studies have revealed several ways to suppress the aggregation processes by different group of molecules or compounds which include metal ions, acridine and its derivatives, polyphenols, plant derivatives, drug molecules and various chemical agents [9,10,11,12,13,14,15]. In all these studies, research has mainly been directed to develop the anti-amyloidogenic or anti-aggregating compounds by studying their effect either on proteins directly involved in neurological and non-neurological disorders [16] or on model proteins having similarity with the them [17,18]. Out of many model proteins, hen egg white lysozyme (HEWL) has been chosen in the present study due to its similarity towards the human lysozyme, a protein associated with the non-neuropathic systemic amyloidosis. It is a monomeric globular protein containing 129 amino acids and comprises of two domains (α and β) cross-linked with four disulfide bridges [19]. Native structure of HEWL is similar to that of the family c-type lysozymes which are naturally occurring glycosidases involved in the degradation of bacterial cell walls [20,21]. The protein interior is almost entirely hydrophobic while both charged amino acid residues and non-polar patches cover its interface.

Small molecules easily intermingled with protein molecules [22,23,24] as well as to the solvent medium that make them the most fascinating agents for inhibiting amyloid formation. One such molecule is D-cycloserine (D-4-amino-3-isoxazolidone), a cyclic structural analogue of D-alanine (D-Ala), chosen in present study as one of the test drug molecule as it act as partial agonist at the glycine recognition site of the glutamatergic NMDA receptor and can boost extinction learning in animals[25]. Furthermore, it also affect non-spatial reference memory in a similar manner as shown for spatial reference memory in rats [26]. Thus, although originally designed to be anti-tuberculosis agent, DCS was later used as a potential treatment for Alzheimer’s dementia and negative symptoms in Schizophrenia [27]. The second chosen anti tuberculosis drug molecule for present study is pyrazinamide (PYZ) which is a nicotinamide analog. Recently, nicotinamide reported as anti-amyloidogenic agent in ex-vivo study [28] and also suppresses tau mediated neurodegeneration in *Drosophila* model [29].

Thus, the aim of the present study was to provide a detailed understanding of the *in-vitro* inhibition mechanism of fibrillation in HEWL influenced by the test small drug molecules i.e., DCS and PYZ with two different functional groups i.e.,–CO and–CONH_2,_ respectively. The kinetics of heat-induced amyloid formation in HEWL and then its attenuation by DCS and PYZ was investigated using various spectroscopic and microscopic techniques as well as molecular docking analysis.

## Materials and Methods

Hen egg white lysozyme (HEWL), Thioflavin T (ThT), 1-anilino 8 naphthalene sulphate (ANS), Congo red, pyrazinamide (cat no. P7136; ≥ 97.5% pure) and D-cycloserine(cat no.C6880; ≥ 98% pure) were purchased from Sigma Chemical Co. (St. Louis, MO, USA). All other reagents used were of analytical grade. Double distilled water was used throughout the study.

### 2.1. Sample preparation

The HEWL was dialyzed in 20 mM sodium phosphate buffer pH 7.4 and its concentration was estimated spectrophotometerically using ε_M_ = 37970 M^-1^cm^-1^[30]. The final concentration of HEWL was kept 10 μM and was incubated for 120 h at 65°C under constant stirring in absence and presence of different concentrations of PYZ and DCS separately. The Aβ-42 was prepared as described in literature [31]. The final concentration of Aβ-42 was kept 25 μM and was incubated for 70 h at 37°C under constant stirring in absence and presence of 500μM of PYZ and DCS separately. For further analysis, aliquots were taken from each set at different time intervals. The solutions of PYZ and DCS were also made in 20 mM sodium phosphate buffer (pH 7.4). Stock solutions for ThT, Congo red and ANS were prepared using $\varepsilon_{412\;nm}^{1\%}$ = 36,000 M^-1^cm^-1^ε_M_^498nm^ = 45,000 M^-1^ cm^-1^and $\varepsilon_{350\;\text{nm}}^{1\%}$ = 5000 M^-1^cm^-1^ respectively [32]. The pH measurements were carried out on Mettler Toledo pH meter (Seven Easy S20-K) model using Expert ‘‘Pro3 in 1” type electrode.

### 2.2. Turbidity measurement

Turbidity measurements of aliquots incubated in absence and presence of drugs were carried on Perkin Elmer Lambda 25 double beam spectrometer at 350 nm in a cuvette of 1 cm path length. The equilibrium data obtained from turbidity measurements was fitted using Sigma plot 12.0 to single exponential equation [33]:

$$A=A_{0}e^{-\Lambda [I]}$$ (1)

where A_0_ and A are the turbidities at 350 nm in the absence and presence of inhibitor, *Λ* is the inhibition constant and [I] is the concentration of inhibitor.

### 2.3. ThT fluorescence spectroscopic measurements

The ThT fluorescence measurements were recorded on Shimadzu 5301PC fluorescence spectrophotometer equipped with water circulator (JulaboEyela) from 460 to 600 nm after excitation at 440 nm. The excitation and emission slit widths were set at 10 nm. Prior to ThT fluorescence measurements, aliquots were supplemented with ThT (10 μM) and incubated for 30 minutes in dark. The obtained data was fitted against sigmoidal curve using equation in sigma plot [34,35].

$$F=F_{i}+m_{i}t+\frac{F_{f}+m_{f}t}{1+e^{-[\frac{t-t_{0}}{\tau }]}}$$ (2)

where F is the fluorescence intensity at time t, and t_0_ is the time to attain 50% of maximal fluorescence intensity. (F_i_ + m_i_t) and (F_f_ +m_f_t) represent the initial base line related to the induction time and finnal constant line, respectively. The apparent rate constant for fibril growth is given by 1/*τ*, and the lag time is calculated by t_o_ -2*τ*.

### 2.4. Fluorescence microscopic measurements

The aliquots (discussed in section 1.3.1) were supplemented with ThT in 1:1 molar ratio of protein to ThT and incubated for 30 minutes in dark. The samples were washed thoroughly and visualized under fluorescence microscope using a 100X oil immersion objective (Zeiss M2 Imager; Zeiss, Göttingen, Germany).

### 2.5. Transmission electron microscopy (TEM)

TEM micrographs of HEWL in absence and presence of DCS and PYZ were taken on Philips CM-10 transmission electron microscope operating at an accelerating voltage of 200 kV. The samples on 200-mesh copper grid covered by carbon-stabilized formvar film. Excess of fluid was removed after 2 min and the grids were then negatively stained with 2% (w/v) uranyl acetate.

### 2.6. Far-UV circular dichroism measurements

Far-UV CD measurements were performed on a JASCO spectropolarimeter (J-815) with a thermostatically controlled cell holder attached to a Peltier with multitech water circulator. Far-UV CD spectra were scanned in the range of 200–250 nm in a cuvette of 0.1 cm path length. Each spectrum was an average of three scans. The percent secondary structure was calculated by K2D3 software.

### 2.7. Congo red binding measurements

Increase in absorbance of Congo red along with red shift on protein interaction, is characteristic feature of amyloid formation [36].Aliquots incubated in absence and presence of drugs were supplemented with Congo red at a molar ratio of 1:1 with protein and incubated for 15 minutes. The absorbance spectra (400–700 nm) of the samples were recorded with a UV-visible spectrophotometer (Perkin Elmer Lambda 25) in a 1 cm path length cuvette.

### 2.8. ANS fluorescence measurements

The ANS fluorescence measurements of aliquots incubated in absence and presence of drugs were performed on Shimadzu 5301PC fluorescence spectrophotometer equipped with water circulator (JulaboEyela). The excitation wavelength for ANS fluorescence was set at 380 nm and the emission spectra were recorded from 400 to 600 nm. Both excitation and emission slits were set at 5 nm. Prior to measurements, aliquots were incubated with 50 fold molar excess of ANS for 30 min in dark.

### 2.9. Steady state fluorescence quenching measurements

All fluorescence measurements were done usingShimadzu 5301PC fluorescence spectrophotometer equipped with water circulator (JulaboEyela) at 25°C. The titration of the drugs (0–200 μM) to 5 μM HEWL solutions was carried out in a dual-path length fluorescence cuvette (10×3.5 mm). Intrinsic fluorescence was measured by exciting protein at 295 nm and emission spectra were recorded in the range of 300–450 nm. The excitation and emission slits width were set at 3 nm. Low concentration of HEWL (5 μM) with absorbance value of ~0.08 was used throughout the fluorescence experiments to minimize the inner filter effect. The data analyzed by according to the Stern-Volmer Equation:

$$\frac{Fo}{F}=\;Ksv\;\left[Q\right]\;+1\;=\;kq\;\tau o\;\left[Q\right]\;+1$$ (3)

where F_o_ and F were the fluorescence intensities in absence and presence of quencher (DCS and PYZ), K_sv_ is the Stern–Volmer quenching constant, k_q_is the bimolecular rate constant of the quenching reaction and τ_0_ the average integral fluorescence life time of tryptophan which is ~10^−8^ sec [37]. Binding constants and binding sites were obtained from Eq 4:

$$Log\;(\frac{Fo}{F}\;-1)\;=\;log\;Kb+\;n\;log\;[Q]$$ (4)

where K_b_ is the binding constant and n is the number of binding sites. The change in free energy was calculated from Eq 5:

$$\Delta G{}^{\circ}=-RTlnK_{b}$$ (5)

where ΔG° is free energy change, ΔH° is the enthalpy change, ΔS° is entropy change, R (1.987 cal mol^-1^K^-1^) is a gas constant and T is the absolute temperature (K).

### 2.10. Molecular docking

The molecular docking study was performed using Autodock 4.2 and Autodock tools (ADT) using Lamarckian genetic algorithm. The crystal structure of lysozyme (PDB id: 6LYZ), Aβ-42 (PDB id: 1IYT) and three dimensional structure of PYZ (CID: 1046) and DCS (CID: 6234) were obtained from Brookhaven Protein Data Bank and PubChem respectively. Chain A of the protein was selected, water molecules and ions were removed and all hydrogen atoms were added. Then partial Kollman charges were assigned to proteins. The proteins were set to be rigid and there is no consideration of solvent molecules on docking. The grid size was set to be 90, 90 and 90 Å along X, Y and Z axes with 0.375 Å grid spacing. The GA population size used was 150. We considered only the minimum energy conformation state of ligand bound protein complex in our study out of ten generated binding modes. Discovery studio 3.5 were used for visualization and for the identification of residues involved in binding.

### 2.11. Cell culture

The SH-SY5Y cells were cultured in DMEM-F12 medium in humidified 5% (v/v) CO2/air at 37°C in 10% (v/v) FBS and 100 U/ml penicillin. Besides, SHSY-5Y (human neuroblastoma cell line), rat pheochromocytoma PC12 cells were used only to validate the cell cytotoxicity of amyloid fibrils. These cells were maintained under DMEM medium supplemented with 5% (v/v) fetal bovine serum (FBS), 10% (v/v) horse serum, and 100 U/ml penicillin in 5% (v/v) CO2/air at 37°C. Both cell lines were kind gift [38].

### 2.12. Cell viability (MTT) assay

MTT (3, (4, 5-dimethylthiazol-2-yl) 2, 5-diphenyltetrazolium bromide) reduction assay was used to measure the cell viabilities of PC12 and SH-SY5Y on addition of aliquots incubated in absence and presence of drugs. MTT undergoes reduction by mitochondrial dehydrogenases to soluble formazan that serves as the indicator of metabolically active cells and inhibition of this reaction is indicative of cytotoxicity [16]. For the MTT reduction assays, protein sample with DCS and PYZ separately were added to the PC12/ SH-SY5Y cells in the 96-well plates for 24 h, and MTT reduction was performed. Cells were seeded at 5,000 cells/well on 96-well plates and incubated for 24 h before the treatment. MTT was added to the culture medium to yield a final concentration of 0.5 mg/ml and incubated for 4 h at 37°C in CO_2_ incubator. Extraction buffer was added and then absorbance was measured at 585 nm after overnight incubation using a micro plate absorbance reader (Bio-Rad Instruments,). The untreated cells were used as controls.

### 2.13. Statistical analysis

All data were presented as mean ± standard deviation from 3 independent determinations. The statistical analysis was made by performing one-way ANOVA for 3 independent determinations. Significance of results was determined as p≤0.01, unless otherwise stated.

## Results and Discussion

### 3.1. Inhibitory Effect of PYZ and DCS on aggregation of HEWL monitored by turbidity method

**Fig 1:**
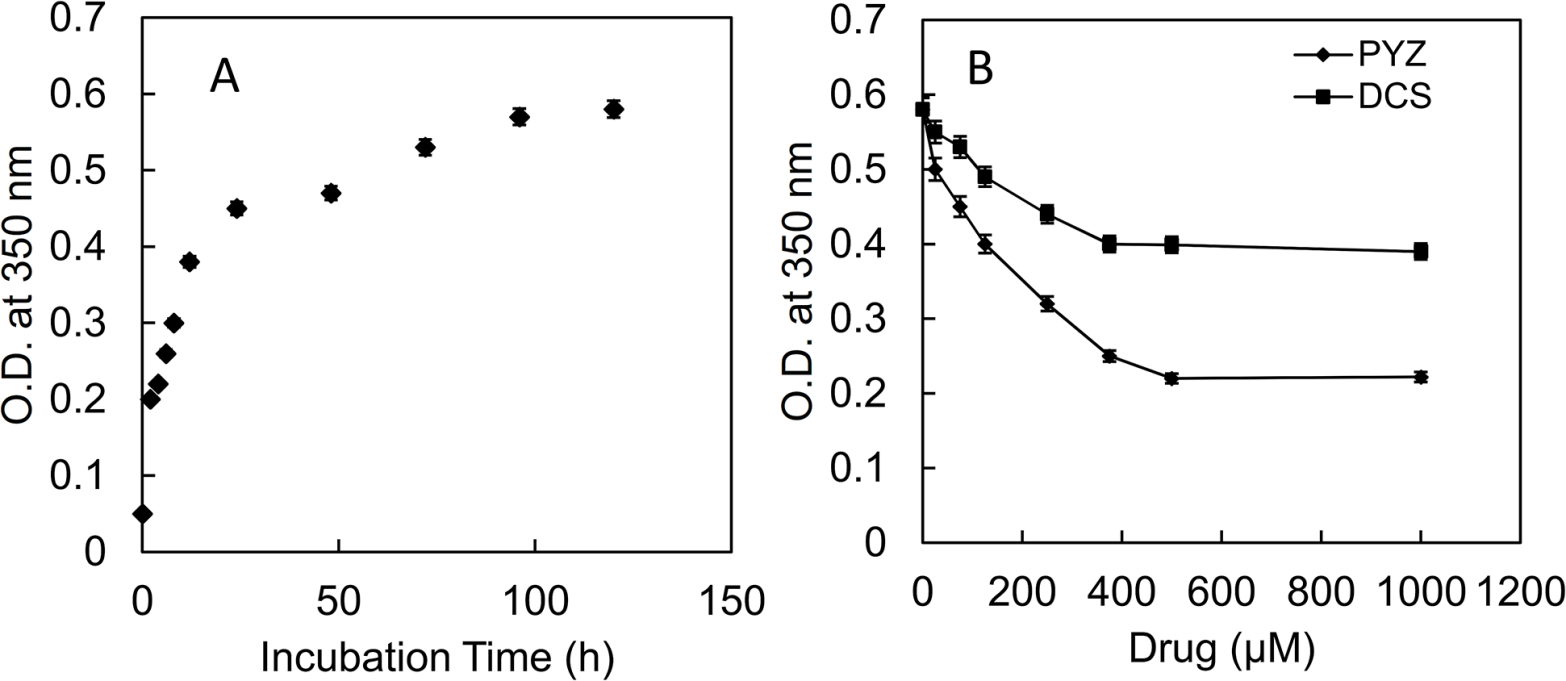
Turbidity measurements by recording O.D. at 350 nm. (A) HEWL incubated at 65°C over a period of 120 h (B) HEWL incubated at 65°C (120 h) in presence of different concentration of PYZ and DCS at 120 h. The maximal concentration of PYZ (1000 μM) and DCS (1000 μM) were taken as control and has negligible O.D. at 350 nm.

As shown in Fig 1A, the turbidity values at 350 nm of HEWL incubated at 65°C at different time increase exponentially with plateau achieved after 72 h. The increase in turbidity values at 350 nm was due to increment in size and number of the aggregates [39]. However, 120 h aged HEWLin presence of varying concentration of DCS and PYZ showed an exponential decrease in turbidity that became saturated at 500 μM of both drugs as shown in Fig 1B. The drop in turbidity value as stated by Zaidi et 2014 al could be attributed to reduction in the size and number of aggregated species [40]. In order to determine the inhibition constant (Λ) of DCS and PYZ for heat-induced aggregation of HEWL, the inhibition curves fitted precisely to single exponential decay equation. The values of inhibition constant (Λ) obtained after fitting for DCS and PYZ are 4.7 × 10^2^ and 1.5 × 10^3^ M^-1^, respectively. It indicated that the PYZ exhibit almost three times higher potential than DCS to inhibit aggregation of HEWL.

### 3.2. Monitoring the anti-amyloidogenic effect of PYZ and DCS by ThT fluorescence measurements

**Fig 2:**
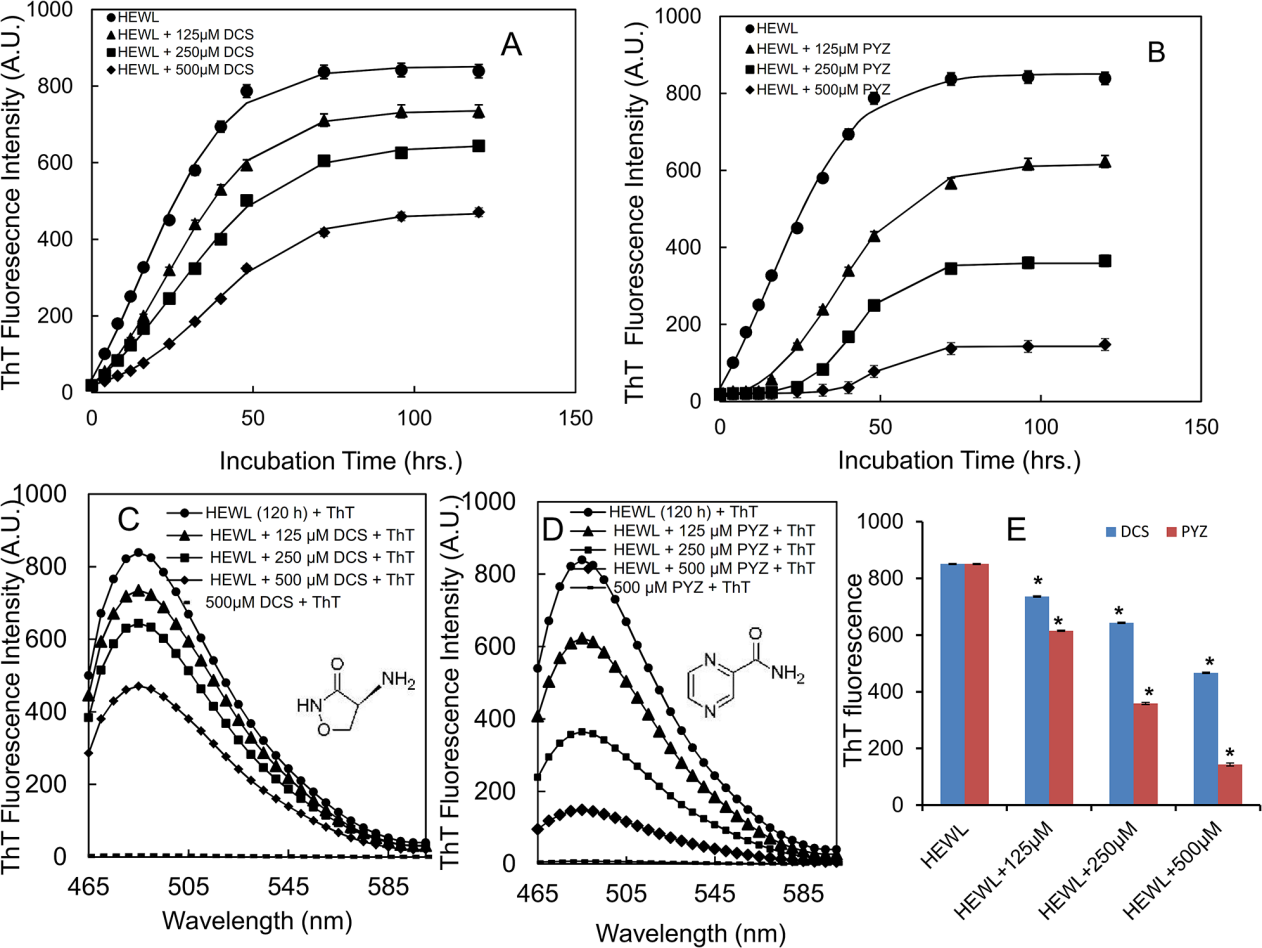
ThT fluorescence kinetics measurements of HEWL. (A) when incubated at 65°C over a period of 120 h in absence and in presence of 125, 250 and 500 μM of DCS (B) when incubated at 65°C over a period of 120 h in absence and in presence of 125, 250 and 500 μM of PYZ. ThT fluorescence spectra of HEWL (C) after incubated at 65°C for 120 h in absence and in presence of 125, 250 and 500 μM of DCS (D) after incubated at 65°C for 120 h in absence and in presence of 125, 250 and 500 μM of PYZ. (E) ThT fluorescence intensity of HEWL (120h) in presence of 125, 250 and 500 μM each of DCS and PYZ. *Statistically different from the control group, p ≤ 0.01 and ^**#**^statistically different from the HEWL, p ≤ 0.01. The 500 μM each of PYZ and DCS were taken as control.

To determine the heat-induced aggregation of HEWL and its inhibition by DCS and PYZ the changes in ThT fluorescence over a period of 120 h in absence and presence of different concentration of DCS and PYZ were individually monitored. Based on turbidity data, three different concentrations (125, 250 and 500 μM) of both drugs were chosen to observed the concentration-dependent aggregation profile of HEWL. The ThT kinetics profile of heat-induced aggregation of HEWL in absence and presence of DCS and PYZ was fitted by using sigmoidal growth equation and results are summarized in Table 1. As shown in Fig 2A and 2B, the time kinetics profile of HEWL (control) exhibited short lag phase of 1.1 h followed by exponential rise till 72 h which get leveled off up to 120 h. It indicated that pattern of aggregation formation was very fast leading to oligomerization and fibril formation. However, elongation in lag time and concomitant reduction in apparent rate constant were observed in presence of increasing concentration of PYZ. The results provided a clear picture that PYZ was slowing down the amyloid formation at the nucleation stage. Similar results were obtained in presence of DCS (Fig 2A). However, the PYZ elongated the lag time many folds with prominent decrease in apparent rate constant (Fig 2B and Table 1). Thus both PYZ and DCS decelerated the amyloid formation however, the PYZ has more pronounced inhibitory effect as it retards the nucleation process. Further, ThT fluorescence spectra of HEWL in absence and presence of DCS and PYZ after 120 h incubation (plateau phase) were compared for determination of potency of inhibitors on aggregation of HEWL (Fig 2C and 2D). The significant reduction in ThT fluorescence intensity observed in presence of PYZ (~82%) and DCS (42%) as can be seen in Fig 2E, Table 1, can be attributed to aromatic interactions between aromatic residues of HEWL and heterocyclic ring of drugs as aromatic stacking interactions are one of the governing factors of amyloid formation [23,24].

**Table 1:** Kinetic parameters obtained on sigmoidal fitting of time kinetics curves and percent reduction in ThT fluorescence intensity of heat induced aggregation of HEWL in absence and presence of varying concentration of DCS and PYZ.

Samples	Lag time (h)	Apparent rate (h^-1^)	% reduction in ThT FI (incubated for 120 h at 65°C
HEWL	1.1±0.01	0.08±0.001	——
HEWL+125 μM DCS	1.2±0.01	0.078±0.001	12.39±1.14
HEWL+250 μM DCS	1.1±0.01	0.076±0.001	23.24±1.79
HEWL+500 μM DCS	7.07±0.03	0.073±0.001	43.86±1.90
HEWL+125μM PYZ	12.24±0.04	0.072±0.001	25.74±1.69
HEWL+250μM PYZ	27.49±0.04	0.06±0.001	57.19±2.11
HEWL+500μM PYZ	38.6±0.05	0.05±0.001	82.24±2.58

### 3.3. Aggregation inhibition visualized by fluorescence microscopic measurements

**Fig 3:**
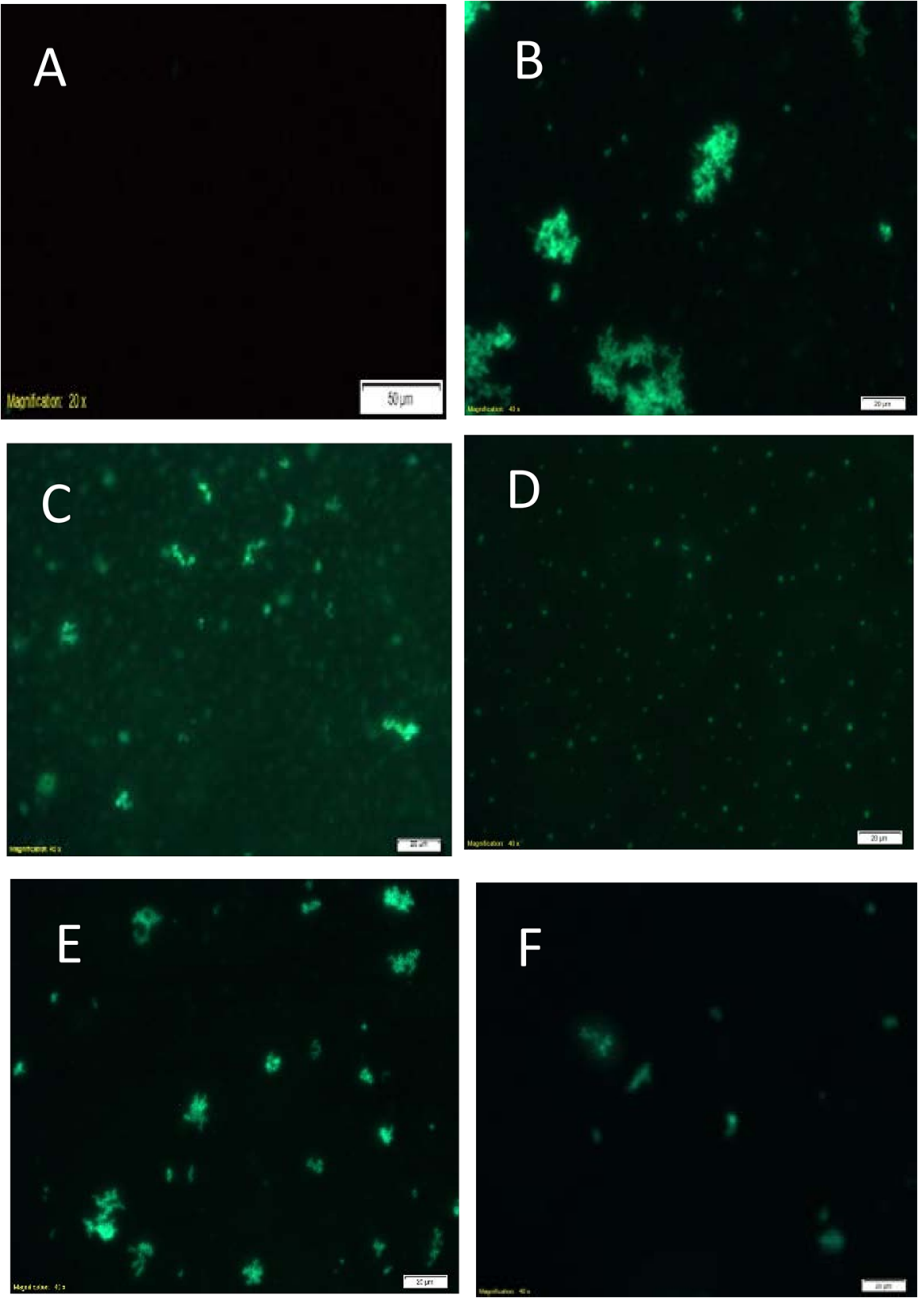
Fluorescence microscopic measurements. (A) HEWL (control) (B) HEWL incubated at 65°C for 120 h (C) HEWL incubated at 65°C for 120 h in presence of 125 μM PYZ (D) HEWL incubated at 65°C for 120 h in presence of 500 μM PYZ (E) HEWL incubated at 65°C for 120 h in presence of 125 μM DCS and (F) HEWL incubated at 65°C for 120 h in presence of 500 μM DCS.

As shown in Fig 3A and 3B, the 120 h aged HEWL upon binding with ThT, fluorescens green which suggested the presence of amyloid fibrils unlike HEWL (control) [41]. The ThT fluorescence reduced in 120 h aged HEWL incubated in presence of 125 μM of PYZ and DCS (Fig 3C and 3D). Furthermore, 500 μM of PYZ led to complete loss of ThT fluorescence whereas similar amount of DCS just diminished the fluorescence (Fig 3E and 3F). It is notable that effect of PYZ was more pronounced as compare to DCS towards aggregation inhibition. These results further confirm ThT fluorescence spectroscopic measurement findings.

### 3.4. Effect of PYZ and DCS on HEWL aggregates examined by transmission electron microscopy (TEM)

**Fig 4:**
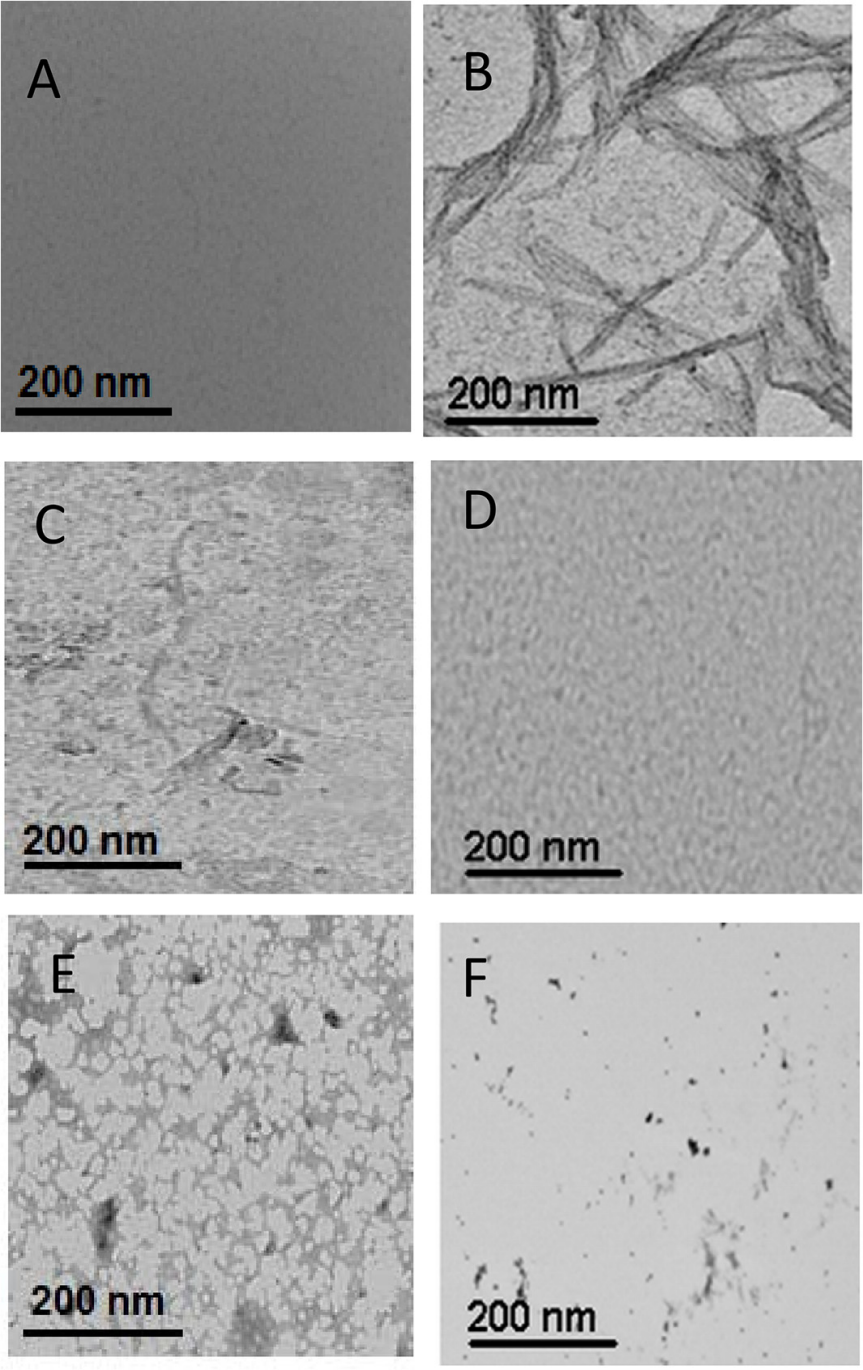
Transmission electron micrographs. (A) HEWL (B) HEWL incubated at 65°C for 120 h (C) HEWL incubated at 65°C for 120 h in presence of 125 μM PYZ (D) HEWL incubated at 65°C for 120 h in presence of 500 μM PYZ (E) HEWL incubated at 65°C for 120 h in presence of 125 μM DCS (F) HEWL incubated at 65°C for 120 h in presence of 500 μM DCS.

Fig 4A–4F, showed the micrographs of HEWL (control), 120 h aged HEWL, 120 h aged HEWL in presence of 125 and 500 μM each of PYZ and DCS. As shown in Fig 4A and 4B, the mature, long straight and dense amyloid fibrils were clearly visible in 120 h aged HEWL unlike control which agrees well with the previous reports [42,43]. However presence of 125 μM PYZ and DCS decreased amyloid fibrillization that got markedly diminish at 500 μM of the two drugs (Fig 4C–4F). Evidently, the inhibition of amyloid fibrillization of 120 h aged HEWL was more pronounced in presence of PYZ which further confirm more potency of PYZ towards inhibition of amyloid fibrillization of HEWL.

### 3.5. Effect of PYZ and DCS on secondary structure of HEWL studied by Far-UV circular dichroism

**Fig 5:**
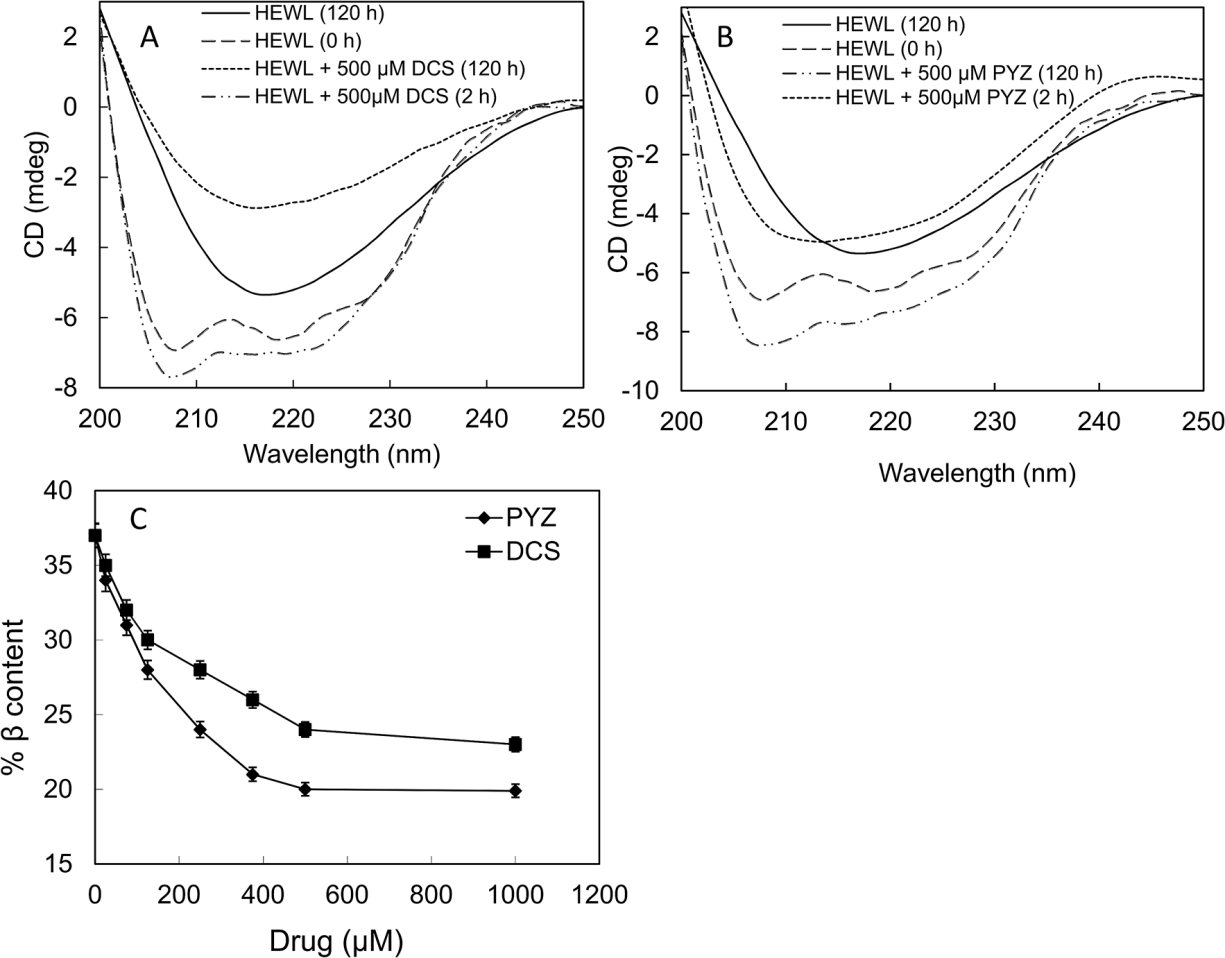
Far-UV CD measurements. (A) Far-UV CD spectra of HEWL incubated at 65°C for different time intervals in absence and presence of DCS (500 μM) (B) Far-UV CD spectra of HEWL incubated at 65°C for different time intervals in absence and presence of PYZ (500 μM). (C) Percent β structure profile of HEWL incubated at 65°C over 120 h with varying concentration of DCS and PYZ (0–1000 μM).

As can be seen from Fig 5 and Table 2, the far UV CD spectrum of the HEWL (0 h) showed higher minima at ~208 nm than at ~222 nm, which is a characteristic of α+β protein (31% α-helix and 11% β-sheet)[44]. After incubation at 65°C for 120 h, the HEWL displayed a structural transition from α-helix to β-sheet conformation (23% α-helix and 37% β-sheet). This structural transition from α-helix to β-sheet conformation indicates the formation of amyloid fibrils in HEWL [17,45]. To know the inhibitory effect of DCS and PYZ, far UV CD measurements of 120 h aged HEWL incubated with varying concentration of both drugs individually were taken (Fig 5A and 5B). The calculated percent secondary structure contents are summarized in Table 2 and Fig 5C. As can be seen from Table 2 and Fig 5C, a decrease in % β-sheet with concomitant increase in % α-helix in HEWL was observed upon incubation with increasing concentration of both DCS and PYZ at 65°C for 120 h. Specifically, the far-UV CD curve of HEWL+500 μM DCS (120 h) (%helix:28; %β sheet:24) has less ellipticity (mdeg) values as compare to the typical curve of HEWL (120 h) (%helix:23; %β sheet:37). The percent secondary structure value along with spectra indicates that DCS have potential to inhibit aggregation but cannot revert protein to its native structure (%helix:31; %β sheet:11). However, far-UV CD curve of HEWL+500 μM PYZ (120 h) (%helix:30; %β sheet:20) have higher minima at ~208 nm than at ~222 nm, which is a characteristic of α+β protein. It indicates that PYZ is potent enough to revert the structure of HEWL (120 h) to a structure which is more close to the native form of HEWL. Thus PYZ and DCS prevented the transition of native α-helix rich HEWL conformer to amyloidogenic β-sheet rich species; however, the PYZ exhibited more pronounced effect. The far-UV CD results further confirm more influential inhibitory effect of PYZ on amyloid fibrillization of HEWL as compare to DCS.

**Table 2:** The secondary structural contents of HEWL incubated in absence and presence of different concentration of DCS and PYZ as calculated from K2D3.

Preparations	% α-helix	% β-sheet
HEWL (0 h)	31±0.20	11±0.10
HEWL (120 h)	23±0.15	37±0.25
HEWL (120 h)+ 125 μM DCS	25±0.16	30±0.20
HEWL (120 h)+ 250 μM DCS	27±0.16	28±0.18
HEWL (120 h)+ 500 μM DCS	28±0.17	24±0.14
HEWL (120 h)+ 125 μM PYZ	25±0.15	28±0.19
HEWL (120 h)+ 250 μM PYZ	28±0.18	24±0.13
HEWL (120 h)+ 500 μM PYZ	30±0.20	20±0.09

### 3.6. Effect of PYZ and DCS on HEWL aggregation examined by Congo red binding assay

**Fig 6:**
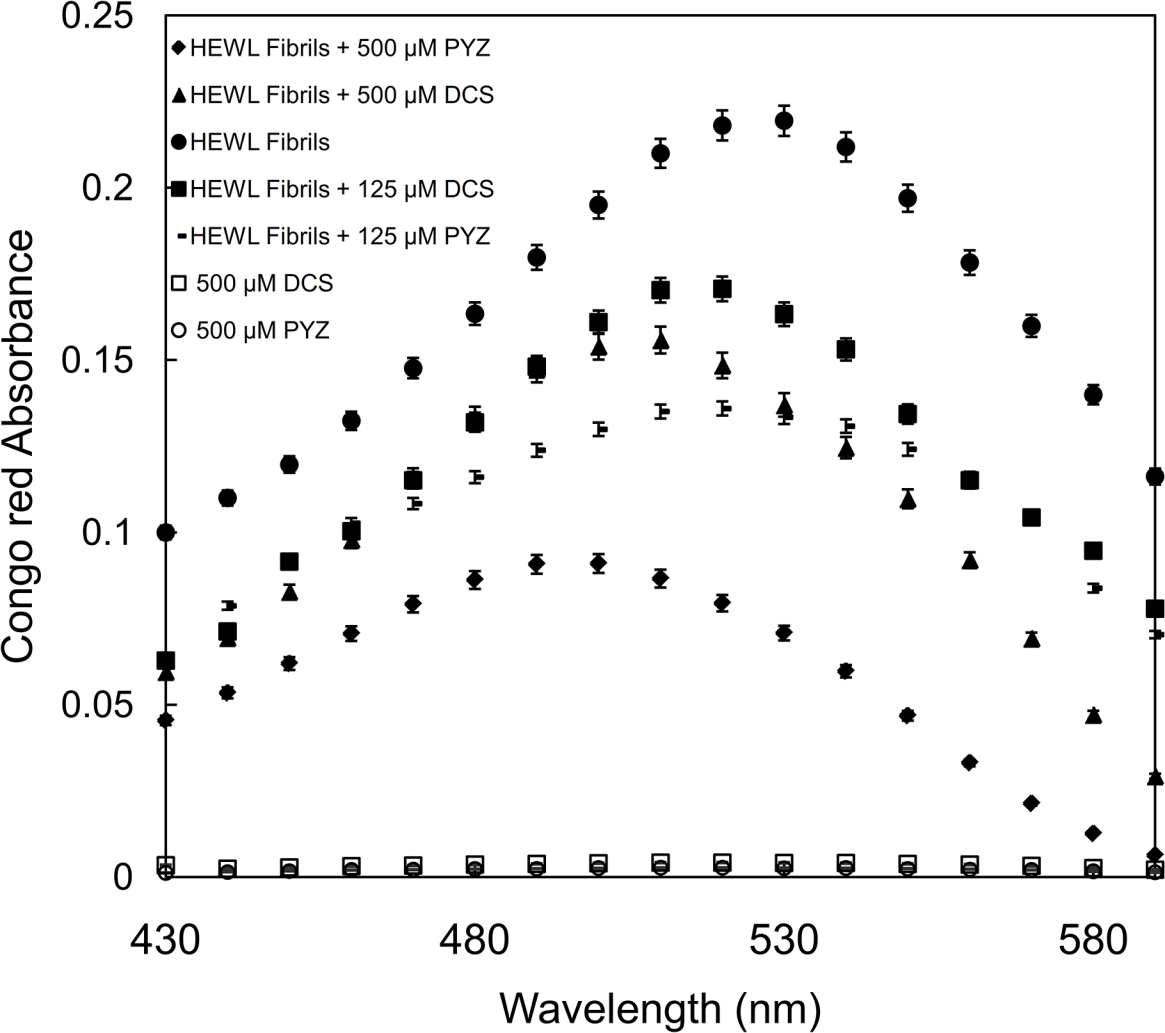
Congo red absorption spectrum of HEWL (10 μM) incubated at 65°C over 120 h in absence and presence of 125 and 500 μM each of PYZ and DCS.

Fig 6 shows an increase in absorbance with absorption maxima at 540 nm. The increase in absorbance with red shift is a characteristic feature of amyloid formation and thus it shows that Congo red binds to matured fibril of HEWL [46]. However, incubation with 500 μM PYZ and DCS led to decrease in absorbance with absorption maxima at around 501 nm and 510 nm along with blue shift of approximate 40 and 30 nm. The decrease in absorbance and shift was concentration-dependent and thus indicate the inhibitory action of drugs against HEWL fibrillation; though more pronounced effect is of PYZ.

### 3.7. Effect of PYZ and DCS on HEWL aggregation by ANS fluorescence measurements

**Fig 7:**
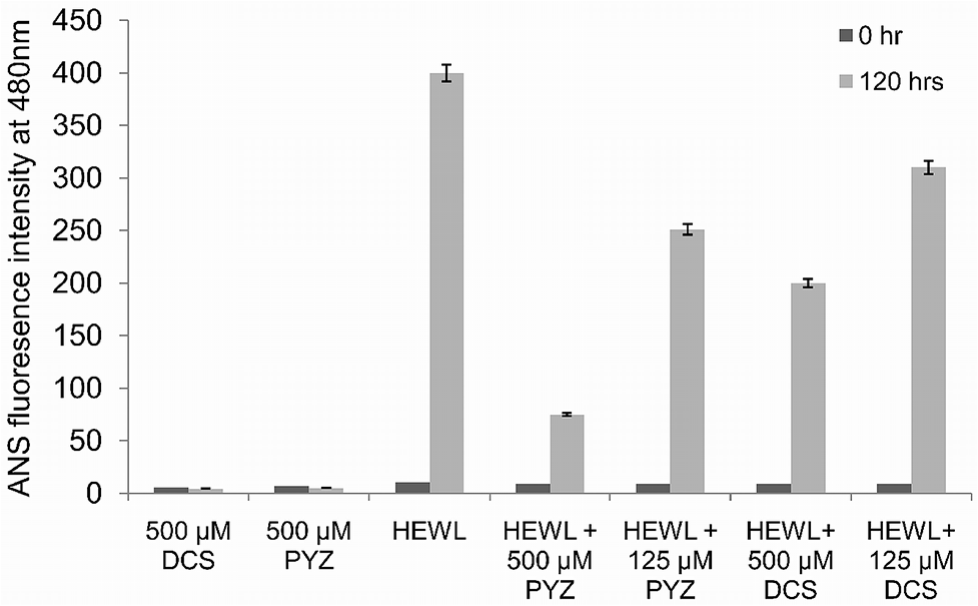
ANS fluorescence intensity measurements of HEWL in absence and presence of PYZ and DCS. The 500 μM each of PYZ and DCS were taken as control.

As shown in Fig 7, the ANS fluorescence of HEWL at zero hour exhibited negligible intensity at 480 nm which confirm the absence of exposed hydrophobic patches. However, HEWL after incubation at 65°C for 120 h resulted in an increase in ANS fluorescence due to exposure of hydrophobic patches as a consequence of amyloid fibrillization.[47] Incubation of PYZ and DCS with HEWL resulted in a significant depreciation of ANS fluorescence in concentration-dependent manner which indicates the reduction in hydrophobic patches exposure. However, the decrement in ANS fluorescence of 120 h aged HEWL amyloid fibrils was more pronounced in presence of PYZ as compared to DCS. It was evident from the Fig 7 that there were around 37.5 and 81% reduction in ANS fluorescence in presence of 125 and 500 μM of PYZ whereas 22.5 and 50% in presence of similar amount of DCS, respectively. This reduction might have occurred due to binding of PYZ and DCS to hydrophobic patches on fibrillar species subsequently reducing their accessibility to bind with ANS.

### 3.8. Binding of PYZ and DCS with HEWL evaluated by steady state fluorescence spectroscopic measurements

**Fig 8:**
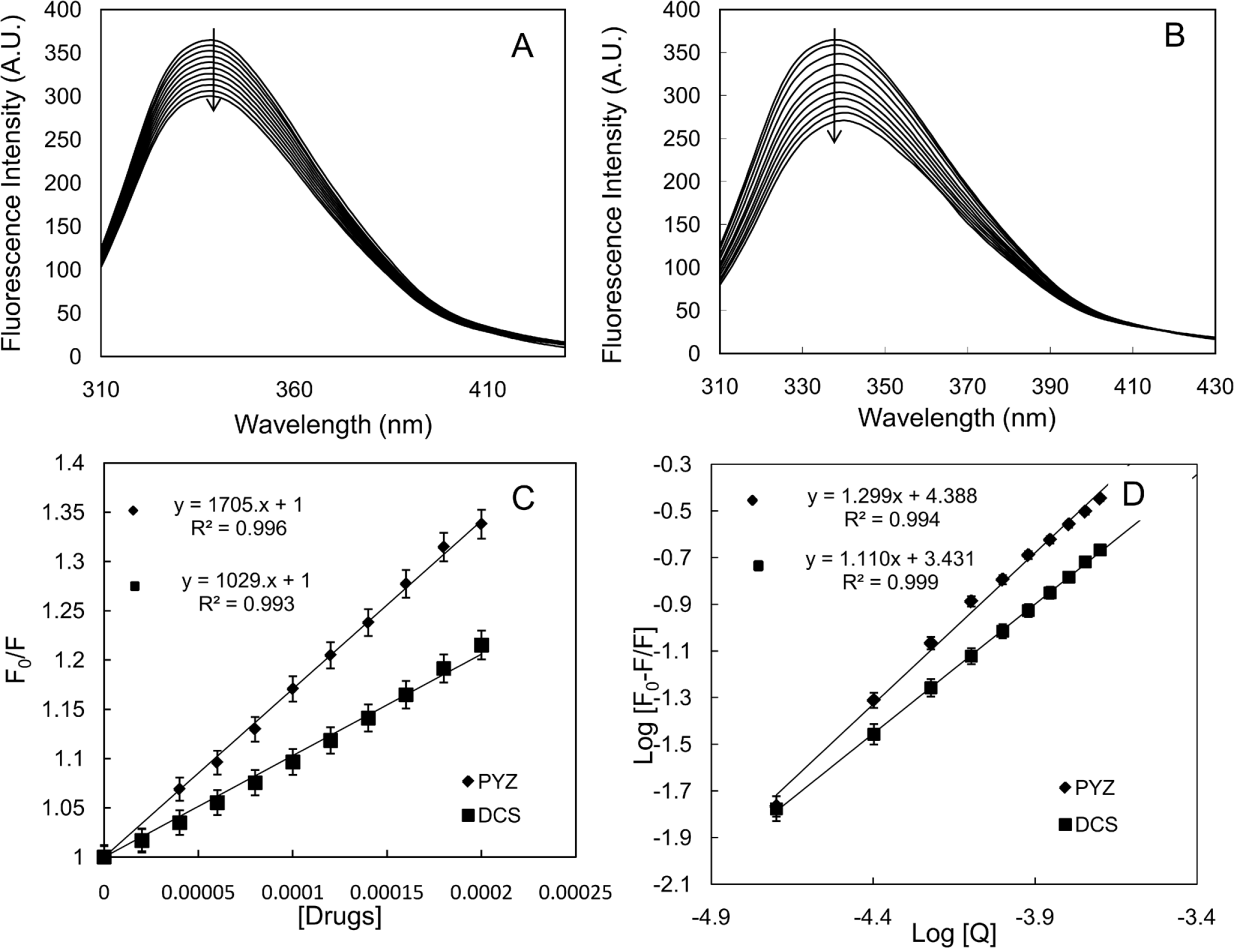
Steady-state fluorescence quenching measurements of HEWL in presence of DCS and PYZ. (A) Fluorescence spectra of HEWL (5 μM) in presence of DCS (0–200 μM) (B) Fluorescence spectra of HEWL in presence of PYZ (0–200 μM) (C) Stern-Volmer curve of HEWL in presence of DCS and PYZ. (D) Modified Stern-Volmer curve of HEWL in presence of DCS and PYZ.

The fluorescence intensity of HEWL at 25°C was taken in absence and presence of varying concentration of PYZ and DCS as shown in Fig 8A and 8B. The fluorescence intensity of HEWL decreased on addition of PYZ and DCS with concomitant blue shift of 1 and 3 nm respectively the in emission maxima. This indicates binding of PYZ and DCS to HEWL as reported earlier [48,49]. In order to get an idea about binding affinity and type of binding, the fluorescence intensity in absence and presence of drugs were analyzed according to the Eqs 3 and 5. The values of *K*_*sv*_, *K*_*b*_, n, and *k*_*q*_ obtained from Stern-Volmer and modified Stern-Volmer plot for both PYZ and DCS (Fig 8C and 8D) are listed in Table 3. It can be seen that the values of *K*_*sv*_ and *K*_*b*_are 1.02×10^3^, 1.7×10^3^ and 2.05×10^3^, 2.45×10^4^ M^-1^ for DCS and PYZ, respectively. Furthermore, the value of *k*_*q*_ was founds to be 10 times greater than the 2×10^10^ M^-1^ s^-1^ (value for maximum scatter collision quenching constant of various quenchers with biopolymers) which confirm that both drugs form complex with the protein [50]. Thus both PYZ and DCS exhibit enough ability to form complex with HEWL by interacting with hydrophobic regions however this ability is higher for PYZ. Further, ΔG values calculated by using Eq 5 were found to be more negative for PYZ as compare to DCS. This suggests more spontaneous binding of PYZ with protein compared to DCS. Therefore, drugs by interacting with hydrophobic regions of HEWL decrease the chance of aggregation which is otherwise favored by increase in hydrophobicity of HEWL. These results are in agreement to that obtained from ANS binding measurements and thus present a plausible reason for inhibition of aggregation of HEWL.

**Table 3:** Binding parameters obtained from steady state fluorescence measurements of both DCS and PYZ interaction with HEWL.

Binding parameters	DCS	PYZ
n	1.1±0.02	1.2±0.03
*Ksv* (M^-1^)	(1.02±0.16)×10^3^	(1.70±0.17)×10^3^
*k*_*q*_ (M^-1^s^-1^)	(1.02±0.16)×10^11^	(1.70±0.17)×10^11^
*K*_*b*_ (M^-1^)	(2.70±0.18)×10^3^	(2.45±0.19)×10^4^
ΔG (kcalmol^-1^)	-4.50±0.02	-5.76±0.03

### 3.9. Molecular docking analysis of drug-HEWL

**Fig 9:**
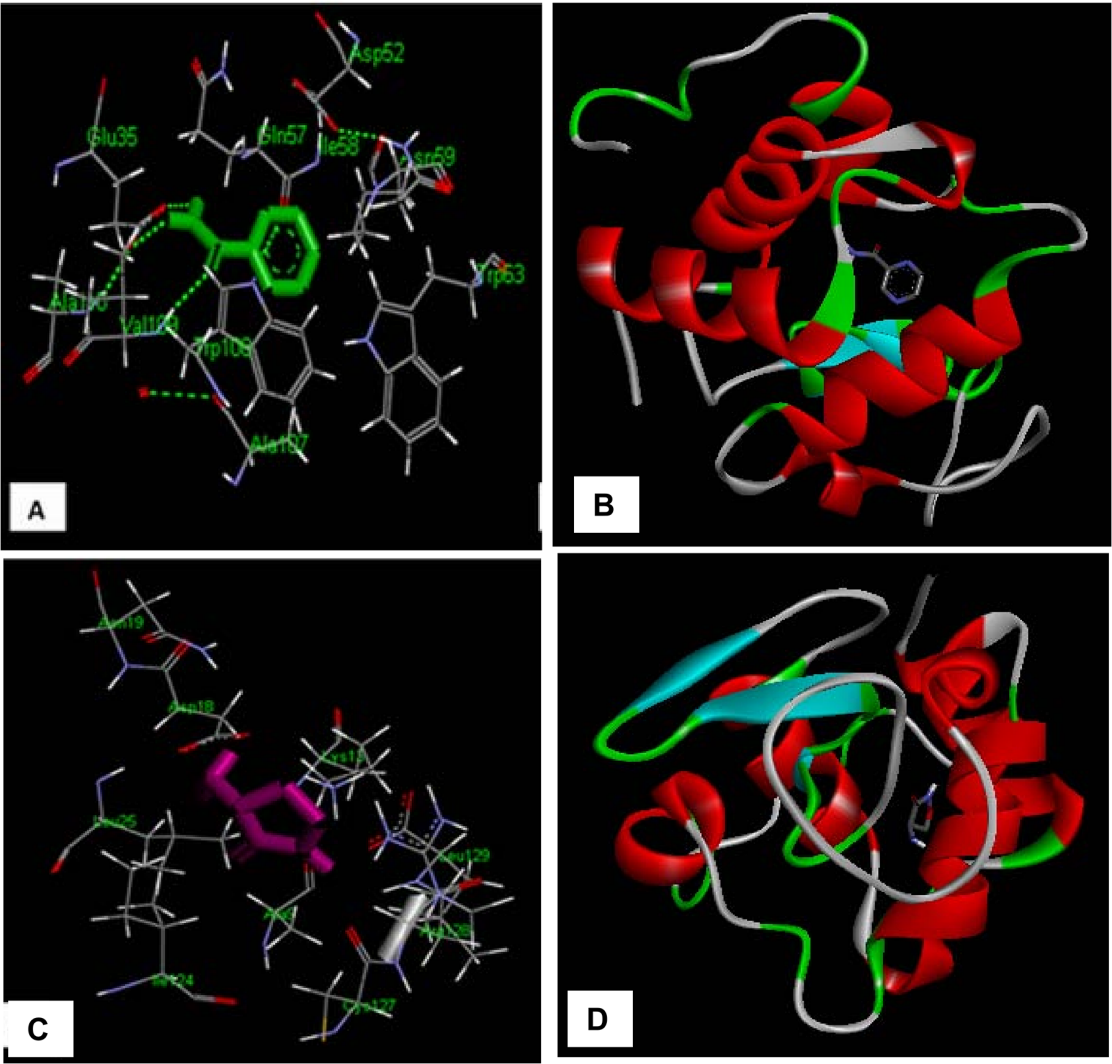
Detailed view of the docking poses of drug [(A, B) PYZ and (C,D) DCS]-HEWL interaction.

As shown in Fig 9A–9D, docking results indicated that PYZ interact with Glu^35^, Gln^57^, Ile^58^, Asn^59^, Trp^63^, Ala^107^, Trp^108^, Val^109^, Ala^110^ whereas DCS interact with Ala^9^, Lys^13^, Asp^18^, Asn^19^ Leu^25^, Ile^124^, Cys^127^,Arg^128^,Leu^129^ (Table 4). As reported in literature [51], HEWL has a stretch of amino acid 54–62 that is highly prone to form amyloid fibrils. Thus PYZ interfere with the amyloid fibril formation as it interacts with HEWL via amino acids (Gln^57^, Ile^58^, Asn^59^, and Trp63) that lie in the highly prone amyloid fibrils forming region. This may be reason of inhibitory potency of PYZ. Moreover, it is reported that Trp^108^ has a key role in stabilization of HEWL[52] and consequently being a part of PYZ-HEWL complex it also support the stabilizing action of PYZ. On contrary, DCS do not interacts with any of the amino acids that lies in amyloid prone region of HEWL but interact with the helical region of HEWL that could be the reason for its less inhibitory potency and stabilization of protein [52]. The docking results also reveal that both hydrogen bonding and hydrophobic interaction are the major contributing forces in the PYZ-HEWL complex formation; however DCS interact mainly through hydrophobic interaction. Hence, molecular docking results support the potent anti-amyloid action of PYZ over DCS.

**Table 4:** Molecular docking parameters of drugs (PYZ/DCS)-protein interaction.

Protein	Drugs	Amino acid residues	Interactions involved	Binding energy (k cal M^-1^)
HEWL	PYZ	Glu^35^, Gln^57^, Ile^58^, Asn^59^, Trp^63^, Ala^107^, Trp^108^, Val^109^, Ala^110^	Hydrophobic and H-bonding	-5.30
HEWL	DCS	Ala^9^, Lys^13^, Asp^18^, Asn ^19^ Leu^25^, Ile^124^, Cys^127^,Arg^128^,Leu^129^	Hydrophobic	-4.70
Aβ-42	PYZ	Val^12^, Gln^15^, Lys^16^, Phe^19^ and Phe^20^	Hydrophobic and H-bonding	-4.74
Aβ-42	DCS	Phe^19^, Phe^20^ and Asp^23^	Hydrophobic and H-bonding	-3.06

### 3.10. Cell viability (MTT) assay

**Fig 10:**
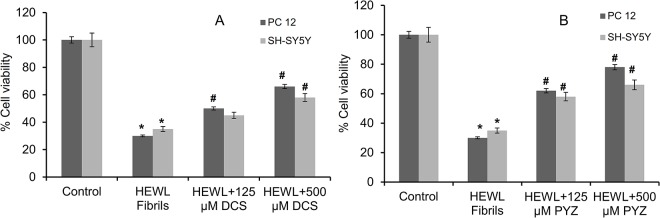
MTT reduction assay for cell cytotoxicity of 120 h aged HEWL amyloid fibrils in SH-SY5Y and PC-12 cell lines in absence and presence of different concentration of (A) DCS and (B) PYZ. Control represents cell lines without prior exposure to HEWL fibrils. *Statistically different from the control group, p ≤ 0.05 and ^**#**^statistically different from the HEWL, p ≤ 0.05 for DCS. *Statistically different from the control group, p ≤ 0.01 and ^**#**^statistically different from the HEWL, p ≤ 0.01 for PYZ.

The aggregated/amyloid fibrils are toxic to neurons; though pre-amyloid fibrils or oligomers are believed to be more cytotoxic [53,54]. Thus, the cytotoxicity of 120 h aged HEWL fibrils were examined on two cell lines i.e., PC-12 and SH-SY5Y by MTT reduction assay at 24 h (Fig 10A and 10B). MTT undergoes reduction by mitochondrial dehydrogenases to soluble formazan that serves as the indicator of metabolically active cells and inhibition of this reaction is indicative of cytotoxicity [38]. As shown in Fig 10A and 10B, the formation of amyloid fibrils with high prevalence of the cross-β-sheet conformation in 120h leads to cytotoxicity and reduced the percent cell viability to around 30 and 35 in PC-12 and SH-SY5Y cell lines, respectively.However, when HEWL fibrils were allowed to form in presence of PYZ and DCS, cell viability increased significantly as compare to that of HEWL fibrils with increase in concentration of both drugs. It may be due to the decrease in cross-β-sheet conformation in HEWL fibrils in presence of both drugs (Table 2). In both PC-12 and SH-SY5Y cell lines the percent cell viability increased to 78%, 66% and 66%, 58% with exposure to lysozyme fibrils in presence of 500 μM PYZ and DCS respectively. Since cross-β-sheet conformation in HEWL is lesser in presence of PYZ as compare to DCS with respect to HEWL fibrils, consequently cell cytotoxicity was affected much by PYZ. Moreover, PYZ and DCS alone in buffer were not showing any significant effect on cell viability. To further exclude the other protective effect of drugs on cell viability; prior to expose with HEWL fibrils, cells were pre-incubated with either PYZ or DCS and no protective effect against HEWL fibrils was found (data not shown). This confirmed that regained cell viability was due to anti-amyloidogenic behavior and ability to decrease cross-β-sheet conformation of drugs.

### 3.11. PYZ and DCS inhibit the amyloid fibrillation of Aβ peptide

**Fig 11:**
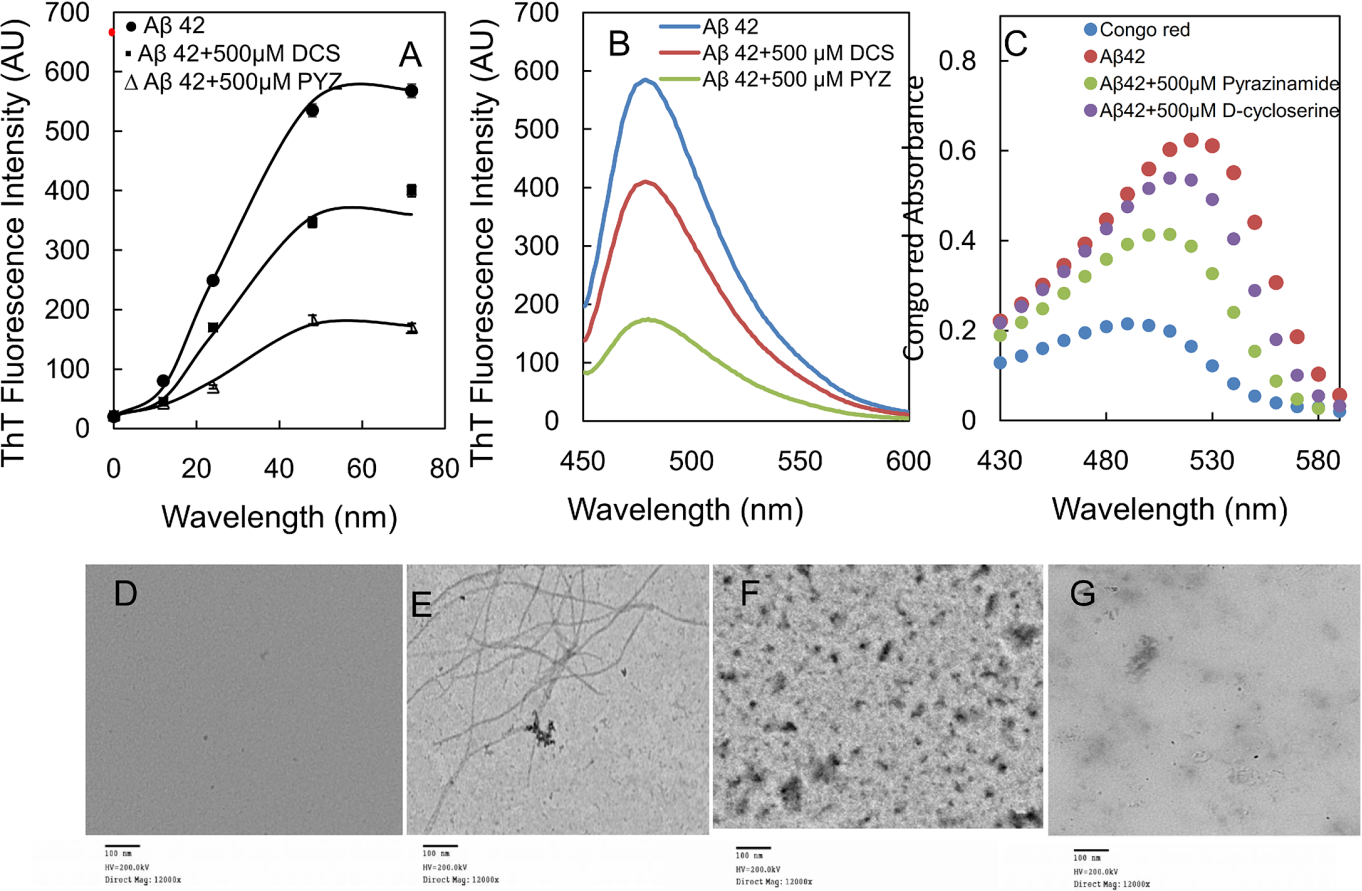
(A) ThT fluorescence kinetics measurements of Aβ-42when incubated at 37°C over a period of 70 h in absence and in presence of 500 μM each of PYZ and DCS (B) ThT fluorescence spectra of Aβ-42 after incubation at 37°C for 70 h in absence and in presence of 500 μM each of PYZ and DCS (C)Congo red absorption spectrum of Aβ-42after incubation at 37°C for 70 h in absence and in presence of 500 μM each of PYZ and DCS, (D) TEM imageof Aβ-42 (E)TEM imageof Aβ-42 incubated at 37°C for 70 h (F) TEM imageof Aβ-42 incubated at 37°C for 70 h in presence of 500 μM PYZ (G) TEM imageof Aβ-42 incubated at 37°C for 70 h in presence of 500 μM DCS.

Fig 11A shows the ThT fluorescence of Aβ-42 in absence and presence of 500 μM each of PYZ and DCS over a period of 70 h. The Aβ-42 binds with ThT and produced increase in the fluorescence whereas incubation of Aβ-42 in presence of 500 μM PYZ and DCS reduced the fluorescence to 70.17 and 36.80% of untreated Aβ-42 respectively as shown in Fig 11B. Similarly, Fig 11C shows an increase in Congo red absorption upon binding with Aβ-42 with absorption maxima at 520 nm along with red shift of 30 nm which indicates the presence of Aβ-42 matured fibril. However, presence of 500 μM PYZ and DCS leads to reduction in absorbance with absorption maxima at around 20 and 25 nm respectively. The reduction in absorbance indicates the inhibitory action of drugs against Aβ-42 fibrillation; though more pronounced effect is of PYZ. These findings are further confirm by TEM micrographs of Aβ-42, 70 h aged Aβ-42, 70 h aged Aβ-42 incubated with 500 μM PYZ and DCS as shown in Fig 11D–11G. The mature and long straight fibrils were clearly visible in 70 h aged Aβ-42unlike control. However presence of 500 μM PYZ and DCS markedly diminish amyloid fibrillization; though PYZ was found to be more effective.

### 3.12. Effect of PYZ and DCS on cell cytotoxicity caused by Aβ peptide fibrillation

**Fig 12:**
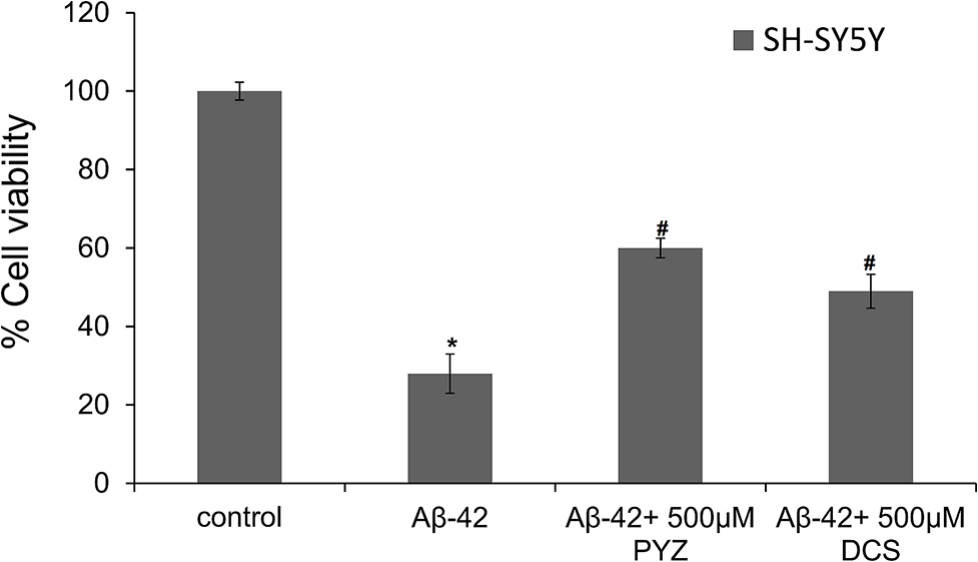
MTT reduction assay for cell cytotoxicity of 70 h aged Aβ-42 fibrils in absence and presence of different concentration of 500 μM DCS and PYZ individually in SH-SY5Y cell lines. Control represents cell lines without prior exposure to Aβ-42 fibrils. *Statistically different from the control group, p ≤ 0.01 and ^**#**^statistically different from the HEWL, p ≤ 0.01.

Fig 12 shows that the 70 hr aged Aβ-42 fibrils leads to cytotoxicity and reduced the percent cell viability to around 30% in SH-SY5Y cell lines. However, when Aβ-42 fibrils were allowed to form in presence of 500 μM DCS and PYZ, cell viability increases to 49 and 60% respectively that further demonstrates more potent effect of PYZ than DCS.

### 3.13. Molecular docking of PYZ and DCS to Aβ-42 peptide

**Fig 13:**
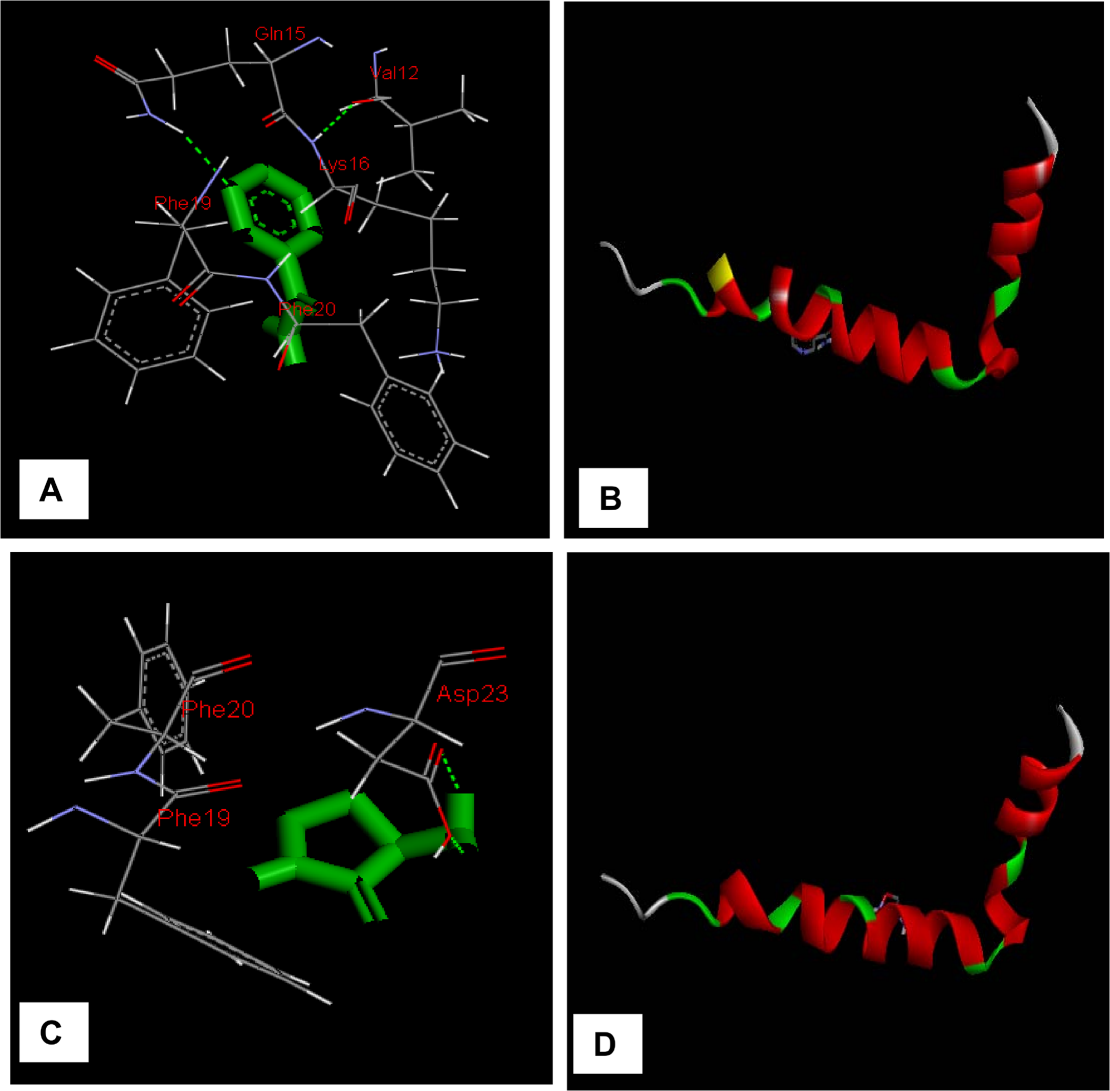
Detailed view of the docking poses of drug [(A, B) PYZ and (C, D) DCS]- Aβ-42 interaction.

The Fig 13A and 13B shows that PYZ interact with five amino acid residues Val^12^, Gln^15^, Lys^16^, Phe^19^ and Phe^20^ of Aβ-42.Among these Val^12^, Gln^15^, Lys^16^ are common residues that are generally involved in the interaction of other inhibitors like Myricetin, EGCG and Curcumin [11]. The PYZ interacts with Gln^15^ of Aβ-42 via hydrogen bond. However, DCS interacts with Phe^19^, Phe^20^ and Asp^23^ amino acid residues as shown in Fig 13C and 13D. Among these amino acid residues the Asp^23^ reported to involve in binding of brazilin via formation of salt bridge [31]. Further, it is found that hydrogen bonding and hydrophobic interaction are the major contributing forces in the DCS-Aβ-42 complex formation.

## Conclusion

The present study established that anti-tuberculosis drugs (PYZ and DCS) also have anti-amyloid potential. Results collectively showed that both drugs inhibit the aggregation in HEWL and Aβ-42 and increase the PC12 and SH-SY5Y cell viability due to concomitant decrease in cross-β-sheet conformation; PYZ being more potent inhibitor than DCS. In contrast to DCS, the presence of heteroaromatic ring with side amino group in PYZ probably makes it more efficient in prolonging nucleation stage of amyloid aggregation through relatively strong binding with amyloid prone region of protein thus disrupting the aromatic stacking and hydrophobic interactions which are involved in amyloid progression. Therefore, it is significantly important to understand the interactions and kinetic parameters for the advancement of therapeutic and diagnostic strategies in conditions in protein aggregation associated diseases.The effect of these drugs are not directly tested on proteins in other amyloid disease like FAP except lysozyme and Aβ-42 (that is believed to be directly involve in Alzheimer’s) but can be implicate to those.
